# Potential Geographic Range of the Endangered Reed Parrotbill *Paradoxornis heudei* under Climate Change

**DOI:** 10.3390/biology12040560

**Published:** 2023-04-06

**Authors:** Wan Chen, Keer Miao, Kun Guo, Weiya Qian, Wan Sun, Hao Wang, Qing Chang, Chaochao Hu

**Affiliations:** 1 College of Environment and Ecology, Jiangsu Open University (The City Vocational College of Jiangsu), Nanjing 210036, China; 2 Jiangsu Key Laboratory for Biodiversity and Biotechnology, College of Life Sciences, Nanjing Normal University, Nanjing 210046, China; 3 Zhejiang Provincial Key Laboratory for Water Environment and Marine Biological Resources Protection, College of Life and Environmental Sciences, Wenzhou University, Wenzhou 325035, China; 4 Nanjing Institute of Environmental Sciences, Ministry of Ecology and Environment, Nanjing 210042, China; 5 Analytical and Testing Center, Nanjing Normal University, Nanjing 210046, China

**Keywords:** bioclimatic, climate change, potential geographic distribution, species distribution model

## Abstract

**Simple Summary:**

The phenomenon of global climate change can impact the geographic range and abundance of species, thereby heightening the vulnerability of rare species to extinction. The reed parrotbill (*Paradoxornis heudei* David, 1872) is endemic to central and eastern China and is considered a near-threatened species according to the International Union for Conservation of Nature (IUCN). This study shows that temperature annual range, annual precipitation, and isothermality were the principal climatic factors to limit the habitat suitability of *P. heudei*. Presently, the suitable habitat for *P. heudei* is predominantly found in the central–eastern and northeast plains of China, especially the eastern coastal area. *P. heudei* is sensitive to climate change. In the future, northeastern China may serve as a potential suitable habitat for *P. heudei*.

**Abstract:**

The phenomenon of global climate change can impact the geographic range and biodiversity, thereby heightening the vulnerability of rare species to extinction. The reed parrotbill (*Paradoxornis heudei* David, 1872) is endemic to central and eastern China, it is mainly distributed in the middle and lower reaches of the Yangtze River Plain and the Northeast Plain. In this study, eight of ten algorithms of the species distribution model (SDM) were used to evaluate the impact of climate change on the potential distribution of *P. heudei* under current and future climate scenarios and to analyze the possible related climate factors. After checking the collected data, 97 occurrence records of *P. heudei* were used. The relative contribution rate shows that among the selected climatic variables, temperature annual range (bio7), annual precipitation (bio12), and isothermality (bio3) were the principal climatic factors to limit the habitat suitability of *P. heudei*. The suitable habitat for *P. heudei* is primarily concentrated in the central–eastern and northeast plains of China, particularly in the eastern coastal region, spanning a mere area of 57,841 km^2^. The habitat suitability of *P. heudei* under different representative concentration pathway (RCP) scenarios was predicted to be different under future climatic conditions, but all of them had a larger range than the current one. The species distribution range could expand by more than 100% on average compared with the current range under the four scenarios in 2050, while it could contract by approximately 30% on average relative to the 2050 range in 2070 under different climate change scenarios. In the future, northeastern China may serve as a potential suitable habitat for *P. heudei*. The changes in the spatial and temporal distributions of *P. heudei*’s range are of utmost importance in identifying high-priority conservation regions and devising effective management strategies for its preservation.

## 1. Introduction

The distribution of species is determined by multiple environmental variables, and many studies have demonstrated that species respond differently to climate change, leading to range adaptations such as expansions, shifts, or contractions to adjust to the new conditions they encounter [1,2,3]. Otherwise, numerous species would be at risk of extinction [4,5]. Climate change has led to substantial changes in the geographic distribution of a large number of taxa, ranging from insects to amphibians and reptiles, mammals, and even birds [6,7,8,9,10]. Studying the transformation of climatic niches throughout geological periods is imperative in comprehending the mechanisms of adaptation, speciation, and extinction, as well as the role of climate in shaping the patterns of species diversity [11]. Previous studies have shown that as a response to global warming, terrestrial species are shifting their distribution towards higher altitudes or latitudes [12]. Predicting the potential habitat in the future and identifying the risk of extinction is essential, as it allows us to take preventive measures beforehand [13]. Understanding the relationship between niche change and climate change can help us understand whether species are capable of adapting their climatic niche to new conditions [14]. When facing changing climatic conditions, lineages with the capability to withstand novel circumstances are chosen, leading to niche evolution through adaptation within the lineages, as well as selective speciation and extinction of various lineages [15]. 

Species distribution modeling (SDM) is a widely used and effective method to assemble and represent the spatial distribution of different taxa [16] using data from observed species distribution records to infer the ecological requirements of species and map their habitat suitability [17,18]; it has been implemented in biological invasion management, the identification and protection of critical habitats, and the selection and migration of protected areas [19,20]. In the past two decades, the use of SDMs has become one of the most effective techniques for investigating the impact of climate change on habitat suitability and the conservation of endangered species, since it does not require field experiments and is easily used to predict distribution under climate change [21,22]. With many different methods and protocols, including generalized linear models (GLMs), generalized additive models (GAMs), MaxEnt (maximum entropy model), random forests (RFs), and bioclimatic envelope, various SDM methods have been employed for the assessment of ecological requirements, ecological responses, and distribution areas [23,24,25,26]. Each model has its own advantages and limitations due to the different principles and algorithms used, and the performance of the model is unstable with the change in input data. Instead of being limited to one model, it is better to set up a model group, integrate the results of multiple models, and take one comprehensive result as the output of the model group to improve the accuracy of the prediction results [27]. Therefore, biomod2, a model platform based on R language, was developed and has been widely recognized and used since its release [1,28].

The reed parrotbill (*Paradoxornis heudei* David, 1872) is mainly distributed in the lower Yangtze River, the Yellow Sea coast of northeastern China, extreme eastern Mongolia, and extreme southeastern Russia and is a resident species endemic to East Asia [29]. Recently, the population of *P. heudei* has been declining due to habitat loss and degradation, and it is listed as near threatened (NT) by the International Union for Conservation of Nature (IUCN) [30]. The reed parrotbill has high habitat specificity and is limited to living in reedbeds, which are very wet areas of reed plants between water and land [31,32]. In the future, this species is facing a moderate-to-rapid decline across its range, mainly due to development and habitat degradation within reedbed habitats. Previous studies have focused on the descriptions of breeding biology, ecology, and genetic markers [31,33,34,35,36]. However, it is unclear how climate change affects population dynamics and where a suitable habitat for *P. heudei* might be in the future. Thus, one crucial issue concerning the ecological importance of *P. heudei* is to ascertain the impact of climate change on the suitable habitat’s geographical distribution. 

Here, we used a large number of geo-referenced records and recent survey data to model the potential distribution of *P. heudei* in China using 10 algorithms and the species distribution model (SDM). In this paper, we hope to use the existing distribution data of *P. heudei* to (1) identify the environmental variables that influence the selection of suitable distribution areas of *P. heudei*, (2) predict the current suitable distribution range of *P. heudei* based on the ecological niche model, and (3) predict the change in the suitable distribution area of *P. heudei* under climate change to provide evidence for the habitat selection and conservation of *P. heudei*.

## 2. Materials and Methods

### 2.1. Species Occurrence Records

Our study area was located in East Asia, mainly in the central–eastern and northeastern regions of China, ranging from 107 to 133° E and from 28 to 49° N based on the distribution range of *P. heudei*. The distribution data of *P. heudei* were collected with field surveys and from the Global Biodiversity Information Facility (GBIF) database [http://www.gbif.org/ (accessed on 13 November 2022)], for a total of 1892 data collected, of which many were duplicate records [37]. Duplicate and invalid records were manually removed; records with obvious misdescriptions of areas and inaccurate descriptions were discarded; and some records without clear geographical distribution were identified and confirmed using Google Earth and GPSspg [http://www.gpsspg.com/maps.htm/ (accessed on 13 November 2022)] for latitude and longitude. To minimize the sampling bias effect in our dataset [38], only one distribution point was randomly retained in a 2.5 arc minute grid cell. After removing points with unclear and incorrect information, we used the spThin package in R 4.0.1 for spatial rarefaction to examine the *P. heudei* records for spatial autocorrelation, which helped to minimize spatial biases and ensure occurrence independence [39]. 

After discarding duplicate and invalid data, we assembled a database of 97 spatially georeferenced occurrence records for model calibration, mainly covering central–eastern China and northeastern China, including Hubei, Henan, Jiangsu, and Heilongjiang Provinces, as well as a few areas of Mongolia and Russia along the Chinese border with China.

### 2.2. Environmental Predictors

Temperature and precipitation data have been identified as the factors that have the greatest impact on the current species niche [40]. We obtained 19 bioclimatic variables at a resolution of 2.5 arcmin using WorldClim [version 2.1; available at https://www.worldclim.org (accessed on 13 November 2022)], which represented the average temperature and precipitation data from 1970 to 2000 [41]. The variables selected included those that represent potential physiological limits for species [42]. To avoid the effect of overfitting, Pearson correlations were used to examine multicollinearity. In the pairwise comparison of the 19 bioclimatic variables, when the absolute value of the correlation coefficient of a certain climatic variable pair was greater than 0.7, it was regarded as a higher contribution, and the corresponding bioclimatic variable was eliminated [43]. 

To measure the changes in species distribution ranges due to climate change, we used four RCP scenarios (RCP2.6, RCP4.5, RCP6.0, and RCP8.5) in two periods, 2050 (average for 2041–2060) and 2070 (average for 2061–2080), with the same spatial resolution of the current period data [44]. Among them, RCP8.5 led to the largest temperature rise, followed by RCP6.0, RCP4.5, and RCP2.6, which had the smallest impact on global warming. An important difference among the four different scenario models is the difference in future land use planning [45]. RCP8.5 is the baseline scenario with no climate change policy intervention; RCP6.0 is the climate scenario with government intervention; RCP4.5 is the climate scenario with another government intervention; and RCP2.6 is the scenario model with very low GHG concentrations.

### 2.3. Modeling Procedure

An ensemble modeling approach with ten algorithms was used to predict the potential distribution of *P. heudei* under current and future climate scenarios; the algorithms were ANN (artificial neural network), FDA (flexible discriminant analysis), GAM (generalized additive model), CTA (classification tree analysis), GBM (generalized boosted model), GLM (generalized linear model), SRE (one rectilinear envelope similar to BIOCLM), MARS (multivariate adaptive regression splines), MaxEnt (maximum entropy model), and RF (random forest), implemented using the biomod2 package in R 4.0.2 software [27]. We obtained 10,000 pseudo-absences or background records for the study area, which are necessary for several algorithms [26]. We employed a five-fold cross-validation method to train the models, whereby 80% of data (including presences and pseudo-absences) were randomly selected for model training, and the remaining data were used for model testing. We used two criterium parameters, true skill statistics (TSS) and area under the receiver operating characteristic curve (AUC) to evaluate the predictive performance of the algorithms [46]. 

To project the habitat suitability of *P. heudei* under current and future climates, the algorithms with TSS ≥ 0.60 and AUC ≥ 0.80 were used for estimating the relative contributions of the predictor variables using a randomization method. To improve the interpretation of distribution changes in current and future climates, we transformed potential distribution projections into binary maps (suitable/unsuitable) by maximizing the TSS value [26,47]. To estimate changes in range size by comparing suitable habitats under current and future climatic conditions, we quantified loss areas, stable areas, and gain areas by counting the number of raster cells (each 2.5 × 2.5 arcmin grid cell) that fell into each category [27]. 

## 3. Results

### 3.1. Predictor Variable Contributions and Model Performance

**Figure 1:**
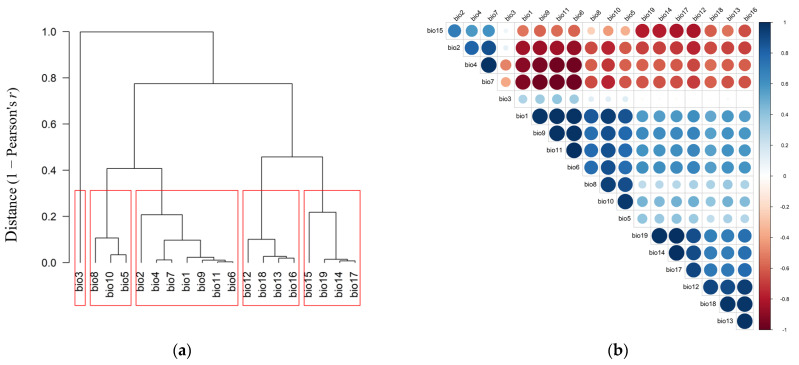
(**a**) Pearson correlations of 19 bioclimatic variables. The variables with correlation coefficients of more than 0.7 were excluded. (**b**) Analysis of 19 climatic factors’ Pearson correlations. The red dots represent positive correlations, and the black dots represent negative correlations. The darker the color and the larger the circle are, the greater the correlation was. If there was no correlation, there is no color.

Based on the results of pairwise Pearson correlation analysis, we chose five climatic variables (isothermality (bio3), i.e., mean diurnal range/ temperature annual range (bio2/bio7); max temperature in the warmest month (bio5); temperature annual range (bio7); annual precipitation (bio12); precipitation in the driest quarter (bio17)) without collinearity to predict the SDM for *P. heudei* in China (Figure 1). 

**Figure 2:**
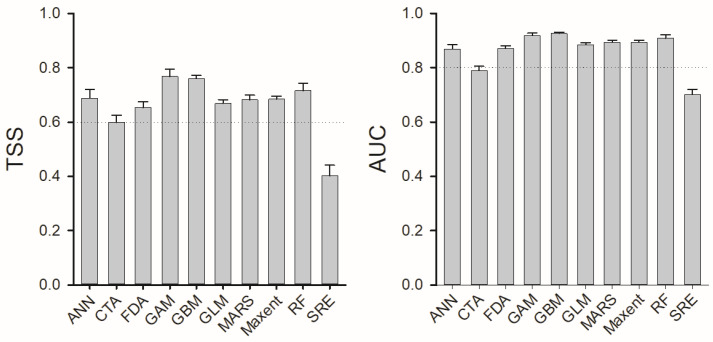
Ten modeling algorithms were evaluated for their predictive performance in estimating the habitat suitability of *Paradoxornis heudei* using the true skill statistics (TSS) and area under the receiver operating characteristic curve (AUC) metrics. ANN: artificial neural network; FDA: flexible discriminant analysis; GAM: generalized additive model; CTA: classification tree analysis; GBM: generalized boosted model; GLM: generalized linear model; SRE: one rectilinear envelope similar to BIOCLM; MARS: multivariate adaptive regression splines; Maxent: maximum entropy model; RF: random forest.

**Figure 3:**
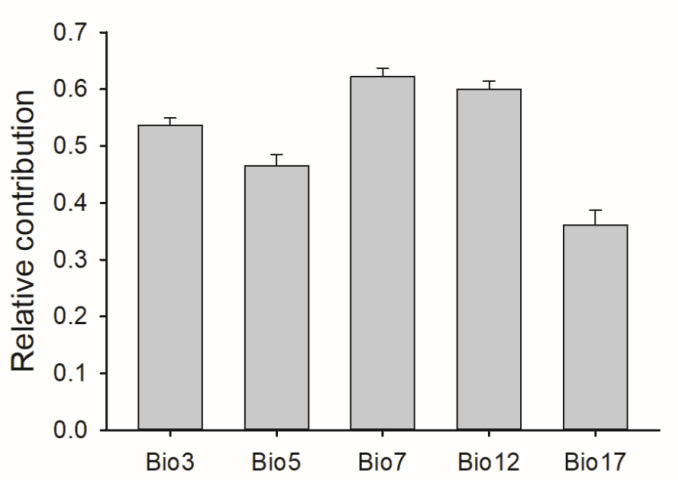
The relative contributions of selected predictor variables in the ensemble model of the habitat suitability of *Paradoxornis heudei* could be determined. bio3: mean diurnal range/temperature annual range; bio5: max temperature in the warmest month; bio7: temperature annual range; bio12: annual precipitation; bio17: precipitation in the driest quarter.

Eight of the ten modeling algorithms showed superior predictive performance (TSS ≥ 0.60 and AUC ≥ 0.80), and these were ANN, FDA, GAM, GBM, GLM, MARS, MaxEnt, and RF (Figure 2). Therefore, these eight models were used to project the habitat suitability of *P. heudei*. According to the basic values of relative contribution, temperature annual range (bio7), annual precipitation (bio12), and isothermality (bio3) were identified as the most important factors influencing the distribution of *P. heudei*. The establishment of *P. heudei* was mainly limited by temperature and precipitation (Figure 3).

### 3.2. Current Potential Distribution

**Figure 4:**
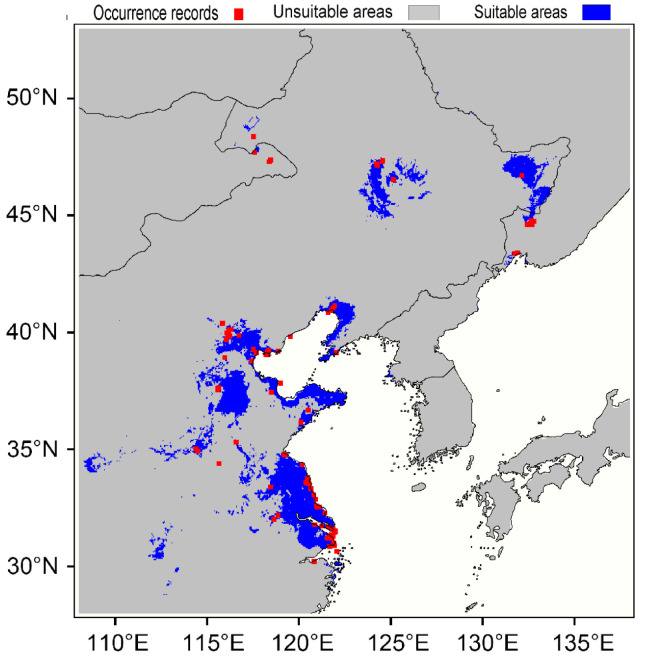
Binary habitat suitability output for *Paradoxornis heudei* based on current climatic conditions. The blue color indicates a suitable range, while the gray color represents an unsuitable range for the habitat. The red squares represent the occurrence records of *Paradoxornis heudei*.

The projection results indicate that the preferred habitat of *P. heudei* was mostly identified in the central–eastern and northeastern plains of China, especially in the coastal areas of the plains. Under the current climatic conditions, the potential habitat area for *P. heudei* covers approximately 57,841 km^2^ and is predominantly located in Northeast Plain, and North China Plain and the middle and lower reaches of Yangtze Plain, with the central area being the most suitable (Figure 4). The suitable habitat for *P. heudei* is extensive but not continuous, exhibiting a fragmented distribution.

### 3.3. Projected Potential Distribution in the Future Considering Climate Change

**Figure 5:**
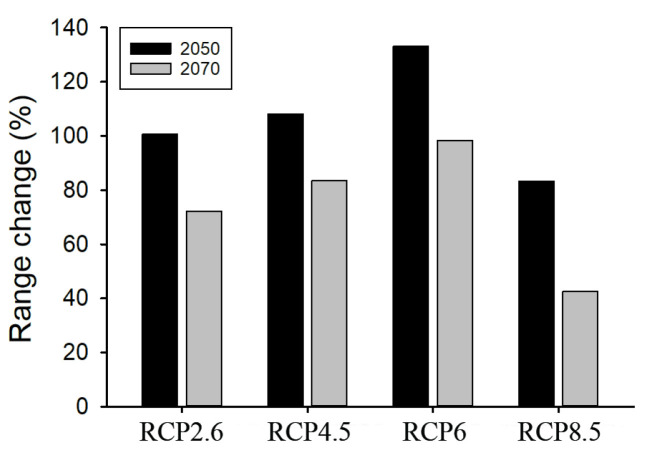
Range size change in *Paradoxornis heudei* in 2050 and 2070 under four RCPs.

Under the four scenarios of RCP6, the projections of the habitat suitability of *P. heudei* under the future climatic conditions were different, but all of them were expanded compared with the current climate range, and the changes were the greatest under the scenario of RCP6. Under this scenario, the suitable distribution area of *P. heudei* could exceed 138,000 km^2^ by 2050, more than double the current area (Figure 5). In both 2050 and 2070, the range could expand and then shrink under different climate change scenarios. In 2050, the range of the four scenarios could increase by more than 100% on average. On average, the 2070 range could be about 30% smaller than the 2050 range.

**Figure 6:**
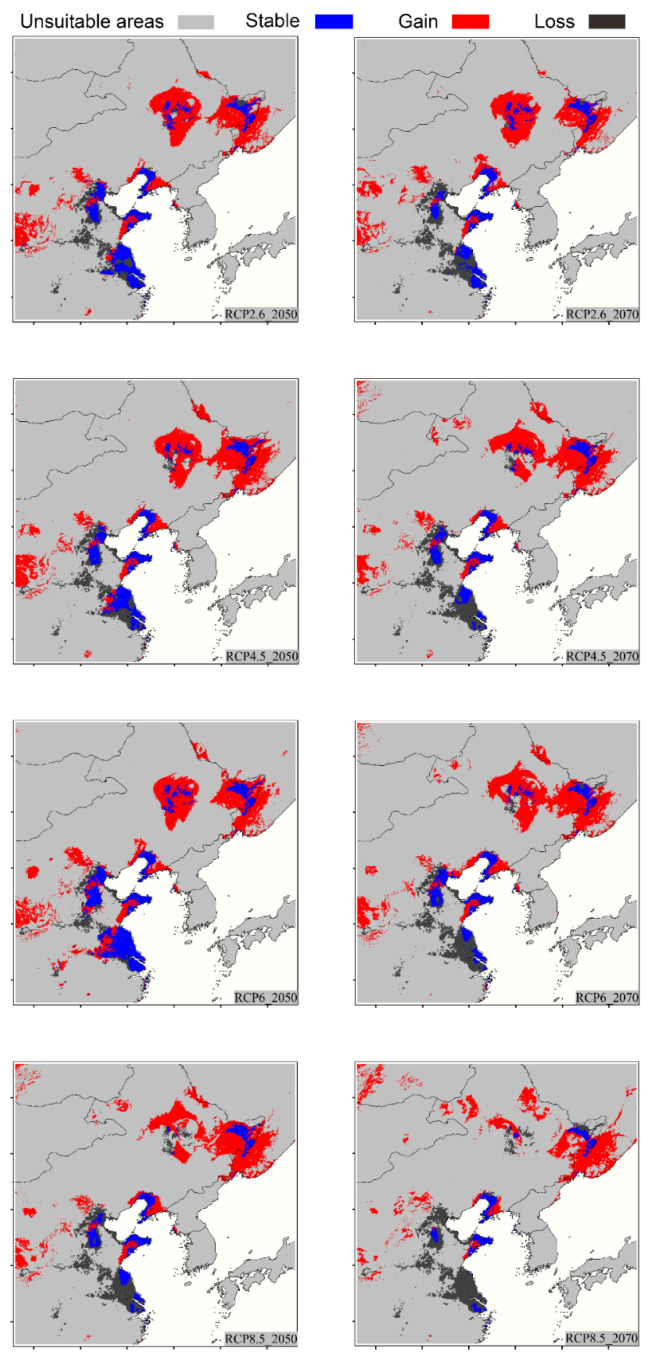
Future distribution of suitable habitats for *Paradoxornis heudei*. The blue areas are currently known to be suitable habitats and may remain so in the future. The red areas represent newly identified suitable habitats projected to emerge in the future. The black areas, which are currently suitable habitats, may become unsuitable due to climate changes.

The simulation results show that the suitable distribution area of *P. heudei* could shift to the north in the future. Compared with the currently suitable distribution area, the expanded area could be mainly concentrated in Songnen Plain, Sanjiang Plain, and Liaodong Bay area in northeastern China, which could become the main habitat of *P. heudei* in the future (Figure 6). However, the suitable distribution area in North China Plain and Jiangsu Province could be significantly reduced in the future, especially in Jiangsu Province, which could be reduced by more than 90% compared with the current range.

## 4. Discussion

In this study, we comprehensively analyzed the potential suitable habitat of the reed parrotbill *Paradoxornis heudei* under present and future climatic conditions, which could serve as a crucial step in developing an effective conservation strategy for the species. Our model indicates that the suitable habitat area for the species under the current climatic conditions covers approximately 57,841 km^2^, mainly in the central–eastern and northeastern plains of China, particularly in the eastern coastal region. This study demonstrates good predictive accuracy and reveals that *P. heudei* is widely distributed. However, the suitable habitats are discontinuous, indicating a fragmented distribution pattern. 

Identifying appropriate variables is a key step to maximize the performance of niche models and their spatiotemporal prediction [48]. Based on the variable importance results, the environmental variable with the highest gain was bio7, indicating that temperature annual range (=bio5–bio6, Max temperature in the warmest month–Min temperature in the coldest month) was the principal climatic factor to limit the habitat suitability of *P. heudei* (Figure 3). The relative contribution rate shows that among the selected climatic variables, annual precipitation (bio12) gave the second highest contribution to the distribution model, indicating that bio12 was an important driving factor for the habitat selection of *P. heudei*. Variables related to extreme environmental conditions, such as bio3 (isothermality) and bio5 (maximum temperature in the warmest month), were found to be important in explaining the distribution. Temperature is a crucial factor in determining the distribution of species, and evaluating the impact of climatic variables across a large geographical area can provide information about suitable habitats for a particular species [49]. Precipitation is also closely linked to wet conditions, such as the amount of water in ponds, streams, and slow-moving wetlands. The reed parrotbill *Paradoxornis heudei* mainly inhabits coastal, lakeside, and riverside wetlands where the common reed *Phragmites australis* is found [32,36]. The results of the species distribution model indicate that the potential suitable habitats for the bird exhibited a fragmented distribution rather than a continuous one (Figure 4). Reedbeds in East Asia are threatened by commercial harvesting, wetland reclamation, and sewage discharge, which have led to a decrease in the quality and size of reed marshes, potentially threatening the survival of *P. heudei*. Temperature and precipitation are considered to be the most predictive variables for birds in the species distribution model [40,50]. 

Under current and future scenarios, the suitability of the habitats showed a large range shift (loss and/or gain), with more habitat suitability towards the northern parts of the study area and some scattered locations (Figure 6). We found that the distribution area of *P. heudei* could be gradually losing their inland habitats and expanding towards coastal areas and the north due to habitat loss. Under different climate change scenarios, the distribution range is expected to expand and then contract in both the 2050 and 2070 time periods. The range is projected to expand by more than 100% compared with the current range under the four scenarios in 2050 but to contract by approximately 30% relative to the 2050 range in 2070. This study revealed that *P. heudei* is sensitive to climate change, which could result in a significant range shift. Northeastern China could be the main distribution areas for *P. heudei* in the future. 

With global warming, some species could induce range shifts and migrate to high latitudes or high elevations, while others may adapt to these changes [51]. We projected the species distribution models into the future to analyze the impact of global warming on the distribution of *P. heudei*. Our predictions suggest that the current trend of global warming could significantly impact the potential distribution of the species, as projected by a simple climate model for the years 2050 and 2070. As temperatures continue to rise, *P. heudei* is expected to shift its range northwards in search of more suitable habitats (Figure 6). Our results showed that with the rise in temperatures, the potential suitable range of *P. heudei* could shift towards higher altitudes and latitudes, gradually moving northward from North China Plain and the middle and lower Yangtze River Plain to Songnen Plain and Sanjiang Plain in northeastern China.

Due to the ongoing climate change, numerous bird species have shifted their geographic ranges towards the north [52,53]. In the past 20 years, the distribution of more than 120 species of birds has expanded northward or shifted westward in China. For example, *Pycnonotus sinensis* Swinhoe, 1870, has spread northward, from the Yangtze River basin and its southern regions to northern China [54]. Sixty-one bird species are expected to shift their cold-zone boundaries poleward by an average of 3.9 degrees by 2070 [7]. Secondly, climate change is also expected to determine major environmental changes in microclimate zones, and potential microclimate zones can support various habitats under relatively stable conditions [55]. Songnen Plain and Sanjiang Plain are encompassed by mountains that block cold air from northern Siberia and water vapor from the ocean. As a consequence, there could be various small areas with unique microclimates and relatively consistent climate and ecological conditions in this region. Nevertheless, microhabitats can lower the mean temperature and the duration of extreme temperatures, thereby mitigating the effects of climate change on species [56]. Slow runoff, seasonal freeze–thaw cycles, and clay and heavy soils all contribute to prolonged surface wetness, excessive water, and poor surface drainage. These conditions eventually lead to the formation of a large marshy water body with multiple saline–alkali lake bubbles and swamp depressions in the affected area. These water bodies have a good role in maintaining temperature, and the annual range of temperature is an important driving factor for the habitat selection of *P. heudei*. According to the findings of this study, *P. heudei* is highly susceptible to the impacts of climate change, which could result in a significant displacement of suitable habitats under future climatic conditions.

The spatial distribution of a species is influenced not only by climate but also by factors such as topography, land cover, human influence, altitude, and other constraints. It is important not to ignore the fact that the habitat of *P. heudei* is unique and heavily reliant on the reed habitat. Therefore, it is suggested that the river system and reed layer be superimposed in the future prediction of the suitable distribution area of *P. heudei*, so as to more accurately simulate the suitable distribution area of *P. heudei* and its future changes. Why the currently suitable habitats in the middle and lower reaches of Yangtze Plain will unsuitable for *P. heudei* in the future, more studies are needed with greater consideration of the ecological requirements.

## 5. Conclusions

In this study, eight of ten algorithms of the species distribution model (SDM) were used to evaluate the impact of climate change on the potential distribution of the reed parrotbill (*Paradoxornis heudei* David, 1872) under current and future climate scenarios. According to the basic values of relative contribution, temperature annual range (bio7), annual precipitation (bio12), and isothermality (bio3) were identified as the most important factors influencing the distribution of *P. heudei*. Under the current climatic conditions, the potential habitat area of *P. heudei* covers approximately 57,841 km^2^ and is predominantly located in Northeast Plain, and North China Plain and the middle and lower reaches of Yangtze Plain, with the central area being the most suitable. The suitable habitat for *P. heudei* is extensive but not continuous, exhibiting a fragmented distribution. The habitat suitability of *P. heudei* under different representative concentration pathways (RCPs) was predicted to vary under future climatic conditions. According to the species distribution model (SDM) predictions, we suggest that the future management strategy for *P. heudei* should consider the potential impacts of climate change on the species and prioritize the conservation of the Songneng and Sanjiang Plain’s wetlands to ensure adequate survival space in the future.

## Data Availability

Environmental data can be obtained from CHELSA [http://chelsaclimate.org (accessed on 13 November 2022)] and WorldClim [http://www.worldclim.org (accessed on 13 November 2022)]. Sampling site information is provided in Table S1.

## Supplementary Materials

The following supporting information can be downloaded at: https://www.mdpi.com/article/10.3390/biology12040560/s1, Table S1: Detailed distributional records of *Paradoxornis heudei* in the present study.

## Abbreviations

ANN: artificial neural network; CTA: classification tree analysis; FDA: flexible discriminant analysis; GAM: generalized additive model; GBM: generalized boosting model; GLM: generalized linear model; MARS: multiple adaptive regression splines; Maxent: maximum entropy; RF: random forest; SRE: surface range envelope; IUCN: International Union for Conservation of Nature; SDM: species distribution model; RCPs: representative concentration pathways; NT: near threatened; GBIF: Global Biodiversity Information Facility; TSS: true skill statistics; AUC: area under the receiver operating characteristic curve.
