# Supplementary material for: Potential Geographic Range of the Endangered Reed Parrotbill Paradoxornis heudei under Climate Change
Source: Biology (Basel). 2023 Apr 6;12(4):560. doi: 10.3390/biology12040560 (PMC10135867; doi:10.3390/biology12040560)
Supplement: Supplementary file 1 [file biology-12-00560-s001.zip › biology-2295230-supplementary.pdf]

Table S1. Detailed distributional records of *Paradoxornis heudei* in the present study.

Species	Order	Latitude (N)	Longitude (E)
Paradoxornis heudei	1	29.97920	121.71600
Paradoxornis heudei	2	30.21000	120.83700
Paradoxornis heudei	3	30.63590	122.06300
Paradoxornis heudei	4	30.85470	121.69000
Paradoxornis heudei	5	30.89100	121.93800
Paradoxornis heudei	6	30.89180	121.90200
Paradoxornis heudei	7	31.00120	121.90700
Paradoxornis heudei	8	31.20920	121.78200
Paradoxornis heudei	9	31.21570	121.54500
Paradoxornis heudei	10	31.29000	121.70800
Paradoxornis heudei	11	31.29730	121.85900
Paradoxornis heudei	12	31.36050	121.78700
Paradoxornis heudei	13	31.46100	121.93000
Paradoxornis heudei	14	31.49960	121.98500
Paradoxornis heudei	15	31.51000	121.96400
Paradoxornis heudei	16	31.60620	121.84900
Paradoxornis heudei	17	31.67360	121.63500
Paradoxornis heudei	18	31.69160	121.58300
Paradoxornis heudei	19	31.72490	121.24300
Paradoxornis heudei	20	31.78790	120.85500
Paradoxornis heudei	21	32.00980	118.64200
Paradoxornis heudei	22	32.18510	118.86300
Paradoxornis heudei	23	32.28270	121.40900
Paradoxornis heudei	24	32.52840	121.10100
Paradoxornis heudei	25	32.53930	121.11500
Paradoxornis heudei	26	32.54420	120.99800
Paradoxornis heudei	27	32.58680	120.96409
Paradoxornis heudei	28	32.86820	120.91200
Paradoxornis heudei	29	33.05420	120.80600
Paradoxornis heudei	30	33.32910	120.66000
Paradoxornis heudei	31	33.40000	118.46667
Paradoxornis heudei	32	33.53900	120.54700
Paradoxornis heudei	33	33.55760	120.42900
Paradoxornis heudei	34	33.58260	120.37700
Paradoxornis heudei	35	33.60230	120.50700
Paradoxornis heudei	36	33.62810	120.53400
Paradoxornis heudei	37	33.63619	120.49100
Paradoxornis heudei	38	33.66670	120.51100

Species	Order	Latitude (N)	Longitude (E)
Paradoxornis heudei	39	33.77663	120.47631
Paradoxornis heudei	40	34.34270	120.18000
Paradoxornis heudei	41	34.40000	115.65000
Paradoxornis heudei	42	34.76690	119.20400
Paradoxornis heudei	43	34.78950	119.25100
Paradoxornis heudei	44	34.93620	114.50600
Paradoxornis heudei	45	35.03333	114.40000
Paradoxornis heudei	46	35.31210	116.59900
Paradoxornis heudei	47	36.15420	120.10300
Paradoxornis heudei	48	36.16800	120.12300
Paradoxornis heudei	49	36.68780	120.50000
Paradoxornis heudei	50	37.45000	118.50000
Paradoxornis heudei	51	37.55650	115.58900
Paradoxornis heudei	52	37.61720	115.59400
Paradoxornis heudei	53	37.64080	115.64400
Paradoxornis heudei	54	37.82920	119.01900
Paradoxornis heudei	55	38.78860	117.41900
Paradoxornis heudei	56	38.93130	115.97800
Paradoxornis heudei	57	39.04330	118.28800
Paradoxornis heudei	58	39.06800	118.21300
Paradoxornis heudei	59	39.12760	117.68400
Paradoxornis heudei	60	39.12830	122.03500
Paradoxornis heudei	61	39.13880	118.28100
Paradoxornis heudei	62	39.17540	118.33700
Paradoxornis heudei	63	39.19740	118.86600
Paradoxornis heudei	64	39.23880	118.34900
Paradoxornis heudei	65	39.27950	117.51700
Paradoxornis heudei	66	39.69100	116.03400
Paradoxornis heudei	67	39.82180	119.52300
Paradoxornis heudei	68	39.83990	116.22700
Paradoxornis heudei	69	39.86620	116.74800
Paradoxornis heudei	70	39.87030	116.19800
Paradoxornis heudei	71	39.89600	116.15900
Paradoxornis heudei	72	39.97244	116.08783
Paradoxornis heudei	73	40.10350	116.18600
Paradoxornis heudei	74	40.13130	116.30800
Paradoxornis heudei	75	40.37790	115.82900
Paradoxornis heudei	76	40.40960	115.84700
Paradoxornis heudei	77	40.85550	121.59502
Paradoxornis heudei	78	40.94340	121.75800

Species	Order	Latitude (N)	Longitude (E)
Paradoxornis heudei	79	41.04350	121.86000
Paradoxornis heudei	80	41.14480	121.95300
Paradoxornis heudei	81	43.37430	131.74600
Paradoxornis heudei	82	43.39888	131.89132
Paradoxornis heudei	83	44.60284	132.68036
Paradoxornis heudei	84	44.61900	132.41200
Paradoxornis heudei	85	44.75020	132.78000
Paradoxornis heudei	86	44.77540	132.65900
Paradoxornis heudei	87	46.52330	125.16000
Paradoxornis heudei	88	46.71290	132.13500
Paradoxornis heudei	89	47.02480	129.05700
Paradoxornis heudei	90	47.14150	124.27161
Paradoxornis heudei	91	47.20090	124.23800
Paradoxornis heudei	92	47.29000	118.38900
Paradoxornis heudei	93	47.30620	124.52400
Paradoxornis heudei	94	47.33310	124.55700
Paradoxornis heudei	95	47.36970	118.45590
Paradoxornis heudei	96	47.68490	117.59200
Paradoxornis heudei	97	48.37310	117.52400
